# Supplementary material for: Influence of Chronic Ocular Hypertension on Emmetropia: Refractive, Structural and Functional Study in Two Rat Models
Source: J Clin Med. 2021 Aug 20;10(16):3697. doi: 10.3390/jcm10163697 (PMC8397123; doi:10.3390/jcm10163697)
Supplement: Supplementary file 1 [file jcm-10-03697-s001.zip › jcm-1326332-supplementary.pdf]

Table S1. (Supplementary Materials): Baseline characteristics of the study.

REFRACTION BASELINE		
35.16 ± 6.38		
**IOP BASELINE (mmHg)**		
13.65 ± 2.49		
**ERG Baseline**		
DA 0.0003 (0.2Hz/s)	a_[ms]	18.85 ± 7.81
	b_[ms]	29.82 ± 12.21
	a_[µv]	32.16 ± 35.36
	b_[µv]	64.41 ± 43.23
DA 0.003 (0.125Hz/s)	a_[ms]	22.24 ± 8.10
	b_[ms]	46.57 ± 7.88
	a_[µv]	30.67 ± 30.23
	b_[µv]	117.32 ± 51.70
DA 0.03 (8.929Hz/s)	a_[ms]	22.57 ± 8.14
	b_[ms]	48.04 ± 3.42
	a_[µv]	72.09 ± 54.91
	b_[µv]	159.69 ± 59.61
DA 0.03 (0.111Hz/s)	a_[ms]	22.84 ± 6.01
	b_[ms]	47.45 ± 3.82
	a_[µv]	53.05 ± 29.51
	b_[µv]	474.35 ± 163.56
PhNR 200microV/div	a_[ms]	15.52 ± 4.93
	b_[ms]	29.78 ± 12.52
	PhNR_[ms]	45.36 ± 5.25
	a_[µv]	36.69 ± 35.72
	b_[µv]	36.69 ± 35.72
	PhNR_[µv]	38.85 ± 28.54

OCT BASELINE (µm)		
RETINAL THICKNESS	CENTRAL	293.28 ± 17.96
	INNER INFERIOR	268.83 ± 9.38
	OUTER INFERIOR	257.67 ± 7.71
	INNER SUPERIOR	262.14 ± 7.91
	OUTER SUPERIOR	260.81 ± 7.75
	INNER NASAL	264.83 ± 9.17
	OUTER NASAL	259.72 ± .65.00
	INNER TEMPORAL	262.83 ± .597
	OUTER TEMPORAL	258.64 ± 6.91
	TOTAL VOLUME	1.87 ± 0.05
RNFL THICKNESS	GLOBAL	49.72 ± 5.61
	INFERIOR TEMPORAL	49.44 ± 11.61
	INFERIOR NASAL	51.75 ± 10.77
	SUPERIOR TEMPORAL	52.14 ± 7.71
	SUPERIOR NASAL	43.03 ± 10.15
	NASAL	48.17 ± 9.78
	TEMPORAL	52.72 ± 8.86
GCL THICKNESS	CENTRAL	22.17 ± 2.75
	INNER INFERIOR	27.50 ± 2.29
	OUTER INFERIOR	25.89 ± 1.98
	INNER SUPERIOR	26.33 ± 2.01
	OUTER SUPERIOR	25.81 ± 2.38
	INNER NASAL	25.97 ± 2.10
	OUTER NASAL	25.97 ± 1.92
	INNER TEMPORAL	26.44 ± 2.30
	OUTER TEMPORAL	26.64 ± 2.04
	TOTAL VOLUME	0.18 ± 0.01

RNFL: retinal nerve fiber layer; GCL: ganglion cell layer; DA: dark adaptation; PhNR: photopic negative response.
